# Mr. Simmons, on the Cæsarean Operation

**Published:** 1799-12

**Authors:** W. Simmons

**Affiliations:** Manchester


### *To the Editors of the Medical and Phyfical Journal.*

Gentlemen,

It was my original intention, in difcuffing the fubject, not to advert to any particular inftance of the Cæfarean operation; but, in confequence of the introduction of my name, and that of Mr. Ogden, into the account of the cafe lately publifhed by Mr. Wood, in the fifth volume of the "Memoirs of the Medical Society of London," I feel myfelf called called upon to ſay a few words in reply. It is the more neceſſary, in the preſent inſtance, as ſome facts are omitted which bear materially on his concluſion; and as the propriety or impropriety of performing this operation muſt reſt on the reſult of well-authenticated caſes, the public have a right to expect the fulleſt evidence upon each of them reſpectively. At the requeſt of Mr. Ogden, I viſited the poor woman, as ſtated by Mr. Wood, and from the information I gained upon an inquiry, it appeared to me to be one of Dr. Osborn's crotchet-caſes; this was the opinion I gave to Mr. Ogden, who thereon determined, for reaſons of a private nature, in which I was perſonally unconcerned, to ſend her to the Lying-in-Hoſpital in Mancheſter; of courſe, no attempt was made to deliver her.

From the manner in which our names are introduced, coupled with his remarks on the caſe, Mr. Ogden and myſelf are compelled to infer, that it was the writer's intention to impute the woman's death to our miſmanagement. Whether this imputation be well founded, the public will now be enabled to determine.

She was conveyed in a cart, as ſtated; but it ſhould have been alſo inſerted, that ſhe was placed on a feather-bed, which was ſlung with cords in imitation of a hammock, by which the ruggedneſs of the road would be counteracted. Mr. Wood's general concluſion is, that "her death "was not occaſioned by the operation, but by a gangrene that had "taken place in the *cervix uteri,* which in my [his] opinion, muſt have "been occaſioned by the preſſure of the child's head prior to the operation; and I am induced to believe, had the operation been performed "earlier, and at the patient's houſe, ſhe would have ſtood a great chance "of recovering."

We are ſtill left to conjecture what time is deemed early enough to give the patient a "great chance of recovering" after this operation; twenty-four hours is uſually deemed the term of a natural labour, when no adventitious help is required. This woman was taken in labour about one o'clock on the Monday morning;—I ſaw her, in company with Mr. Ogden, at nine;—ſhe arrived at the Lying-in-Hoſpital about one; and the operation was performed at nine the ſame evening. The delay then occaſioned by her removal was *four* hours;—the time that elapſed after her arrival at the Lying-in-Hoſpital, before the operation was performed, was *eight* hours;—and from the commencement of labour to the time of the operation was *twenty* hours.

If

If inflammation had exifted prior to the operation, as Mr. Wood has ftated, it muft have been marked by the ufual fymptoms; Mr. Ogden and myfelf are certain that it did not exift at the time we faw her, both from the calm ftate of her pulfe, and the perfect intermiffion between the pains. No notice is taken by Mr. Wood of the ftate of the pulfe prior to the operation; nor is it obferved that the pains had any peculiar character, which muft have been the cafe, had inflammation exifted from the caufe alledged; it is fimply ftated, that they were *very frequent*.

But, if inflammation of the uterus had come on after we faw her, yet previous to the operation, how comes it that it was not difcovered? The day after the operation, fhe was thought to be in no danger; and twenty-five hours from the time of performing it, her pulfe beat only 108 ftrokes in a minute. The following morning, we find that the pulfe had increafed to 120; at noon of the fame day it beat 144; and on Thurfday morning, at feven o'clock, it beat 150. Thus moft diftinctly marking the progreffive increafe of the difeafe, as caufed by the operation; for it is impoffible that the pulfe fhould have continued at fo low a number, for fo long a time, if inflammation of the uterus had exifted prior to it.

Gangrene is faid to have been difcovered, on diffection, in the "inferior portion of the body and *cervix uteri*." If it had been occafioned by the preffure of the child's head, as afferted, it muft have had an appearance correfponding to the form of the body which produced it; but this circumftance, which would have been almoft decifive of the caufe, is not alluded to. That it fhould have been occafioned by the preffure of the child's head, is, indeed, extraordinary. In other inftances, the head fhall be jammed in between the bones of the pelvis, for twice the length of time that elapfed from the coming on of labour to the performance of this operation, without any material inconvenience enfuing to the mother after her delivery. In this cafe, in Mr. Wood's opinion, and I fhould be forry to mis-ftate it,—the intermitting contractions of the uterus, preffing the child's head againft a part of its own body at each contraction, which was again refifted by the foft, elaftic fubftance of the abdominal mufcles, brought on inflammation, terminating in gangrene, though fuffered to continue but for a few hours. It fhould be remembered, too, that the upper part of the head of a child, at the time of birth, is compofed of diftinct bones fo loofely connected together as to admit of being lapped over each other when compreffed, and yet fo elaftic as foon to recover its original fhape on removal of the preffure, which which muſt materially leſſen the chance of miſchief from its acting mechanically. And from the poſition of the child in utero, and the peculiar curvature of the ſpine, it is highly probable that the mere weight of the lower parts of its body, would retract the head from preſſing againſt the uterus, at each interval between the pains. Yet this was the part, according to Mr. Wood, that by its preſſure for ſo ſhort a time, produced gangrene of the uterus. It is obvious, from the extreme narrowneſs of the pelvis, that the head could not deſcend, ſo as to preſs the uterus againſt any of the bones of which it is compoſed. Mr. Wood does not mention that the natural ſhape of the head was at all changed.

In performing the operation, Mr. Wood made an inciſion through the common integument and abdominal muſcles, to the extent of ſix inches; he then made a correſponding inciſion through the body of the uterus. An inciſion of the nature of the former, as largely expoſing the cavity of the abdomen, has been uſually deemed dangerous; and a large wound of the uterus has been commonly looked upon as mortal. The diſeaſed appearances obſerved in the abdomen prove the exiſtence of peritoneal inflammation, and of inflammation of the inteſtines; ten or twelve ounces of bloody ſerum were found extravaſated into the cavity, together with ſome coagulated blood. By referring the cauſe of her death to inflammation of the uterus, terminating in gangrene, and cauſed as abovementioned, Mr. Wood has regarded theſe appearances as of little moment, though peritoneal inflammation, or inflammation of the inteſtines, when ſingly exiſting, proves frequently mortal, even when not attended with any extravaſation of blood.

The quantity of blood loſt during the operation was about eight ounces; how it was diſpoſed of, he does not tell us; neither does he mention how long the cavity of the abdomen was expoſed; nor whether the epigaſtric artery was divided in the operation, which is probable from the direction of the inciſion; yet theſe are points which ſome may think material, as tending to aſcertain the real cauſe of her death.

But admitting that inflammation exiſted in the uterus prior to the operation, let us next inquire into the propriety of the after-treatment, under theſe circumſtances of accumulated danger. In inflammation of the uterus, as well as of the inteſtines, the diſeaſe is moſt powerfully combated by bleeding, which is directed to be employed in ſuch caſes, as far as the conſtitution of the patient and ſtrength of the pulſe will bear. It is obſerved that the pulſe was *hard* at different periods of the diſeaſe, difeafe, yet both general and topical bleeding were entirely omitted. In inflammation of the uterus, and of the inteftines, the frequent injection of clyfters is generally infifted on. In this cafe, though inflammation of the uterus is faid to have exifted at the time of the operation, the firft clyfter was not injected fooner than feventeen hours; the fecond, not till Wednefday noon, after an interval of thirty-nine hours; and forty-eight hours had elapfed from the time of the operation, when the bliftering plaifter was directed to be applied to the abdomen. Indeed, it does not appear that the precaution of injecting a clyfter previous to the operation had been attended to, though the woman had been then eight hours in the hofpital.

Upon the mature confideration of the above, which are the leading circumftances of this cafe, the profeffional reader will be enabled to decide upon the probable caufe of the patient's death; and whether it was occafioned by gangrene of the uterus, brought on by preffure of the child's head prior to the operation; or what greater chance for fuccefs there would have been, had the operation been performed earlier, and at her own houfe.

Mr. Wood's opinion pre-fuppofes little danger to attach to the operation itfelf, as her recovery would, in that cafe, have been confidently reckoned upon. A large incifion made into the uterus and its confequences, extravafation into the cavity of the abdomen, peritoneal inflammation, and inflammation of the inteftines, are as confidently rejected, as being infufficient to account for her death.

Upon fuch inconfequent reafoning is this operation to be perfifted in? an operation which, in my opinion, is *in itfelf mortal*, and which has certainly proved mortal in this country in every inftance. When a queftion is to be decided by numbers, the voice of an individual will be of trivial import; but whether this operation will be permitted to be performed in oppofition to reafon and fact, time will fhew.

Thefe obfervations would have been more properly placed in the volume containing Mr. Wood's account; but as that is now impracticable, I am perfuaded, from the zeal to promote the true interefts of the profeffion which you have manifefted, and from your candour and impartiality, that you will give them infertion in an early number of your publication.

